# Validation of conversion between mini–mental state examination and montreal cognitive assessment

**DOI:** 10.1002/mds.26498

**Published:** 2016-02-10

**Authors:** Michael Lawton, Meike Kasten, Margaret T. May, Brit Mollenhauer, Martina Schaumburg, Inga Liepelt‐Scarfone, Walter Maetzler, Eva‐Juliane Vollstedt, Michele T.M. Hu, Daniela Berg, Yoav Ben‐Shlomo

**Affiliations:** ^1^ School of Social and Community MedicineUniversity of BristolBristolUnited Kingdom; ^2^ Department of Psychiatry and Psychotherapy and Institute of NeurogeneticsUniversity of LübeckLübeckGermany; ^3^ Paracelsus‐Elena‐KlinkKasselGermany; ^4^ Departments of Neurosurgery and NeuropathologyUniversity Medical Center GöttingenGermany; ^5^ German Center for Neurodegenerative Diseases (DZNE)TuebingenGermany; ^6^ Department of Neurodegeneration, Hertie Institute for Clinical Brain Research (HIH)University of TuebingenTuebingenGermany; ^7^ Nuffield Department of Clinical Neurosciences, Division of Clinical NeurologyUniversity of OxfordUnited Kingdom; ^8^ Oxford Parkinson's Disease CenterUniversity of OxfordOxfordUnited Kingdom

**Keywords:** Parkinson's disease, Mini–Mental State Examination, Montreal Cognitive Assessment, equating

## Abstract

**Introduction:**

Harmonizing data across cohorts is important for validating findings or combining data in meta‐analyses. We replicate and validate a previous conversion of MoCA to MMSE in PD.

**Methods:**

We used five studies with 1,161 PD individuals and 2,091 observations measured with both the MoCA and MMSE. We compared a previously published conversion table using equipercentile equating with log‐linear smoothing to our internally derived scores.

**Results:**

Both conversions found good agreement within and across the studies when comparing true and converted MMSE (mean difference: 0.05; standard deviation: 1.84; median difference: 0; interquartile range: –1 to 1, using internal conversion).

**Conclusions:**

These results show that one can get a reliable and valid conversion between two commonly used measures of cognition in PD studies. These approaches need to be applied to other scales and domains to enable large‐scale collaborative analyses across multiple PD cohorts. © 2016 International Parkinson and Movement Disorder Society

There are many studies of individuals with Parkinson's disease (PD), although often data are limited by selected samples and small sample sizes meaning that analyses are underpowered. Researchers often seek to validate previous research in a new data set or combine data in a meta‐analysis, but comparing and pooling findings may be problematic if different measures have been used. A common nonmotor feature of PD is cognitive impairment, and two of the most popular screening tools are the Mini–Mental State Examination (MMSE)^1^ and the Montreal Cognitive Assessment (MoCA).^2^ There are well‐established methods for scale conversion^3^ and a previous study^4^ has applied these to convert the MoCA to the MMSE in patients with PD. The validity of this conversion has been evaluated in a small sample of 139 subjects with PD, with a narrow distribution of MoCA scores, which suggested it was reasonably good.^5^ We present both a replication of the methods of van Steenoven and colleagues and a validation of their conversion chart in a much larger independent sample including individuals with a wider range of MoCA and MMSE scores.

## Patients and Methods

### Study Population

We used data from five studies that are a part of the Joint Programme Neurodegenerative Disease (JPND) consortium who have data collected on both the MMSE and MoCA in patients with PD. In all cases, the MoCA scores were adjusted by adding 1 point (to a maximum of 30) for all those with 12 years or less of education. Brief details are provided below.

The MODEP cohort consists of incident PD (n = 22), prevalent PD (n = 18), and controls (n = 24) with up to 8 visits.^6^ We restricted this analysis to the incident and prevalent PD arms. The ABC‐PD study is an ongoing cross‐sectional study of PD with 91 individuals included in this analysis. The De Novo Parkinson (DeNoPa) study is an untreated incidence PD cohort (n = 159) with a control arm (n = 110).^7^, ^8^ This cohort has data at baseline and on one follow‐up visit. We included 123 PD patients after excluding individuals who were found to have other neurological diseases at follow‐up. The EPIPARK cohort has 112 PD patients and 543 controls with data on up to three visits.^9^ We restricted this analysis to the PD patients. The Oxford Discovery cohort has data on 958 PD patients, 293 controls, and 180 at risk of PD (as of January 2015)^10^ with data on up to three visits per individual. We included only the 795 PD patients who were recently diagnosed (within 3.5 years) and with clinical probability of PD ≥90%.

The analyzed data included only the PD subjects for direct comparison with the original van Steenoven and colleagues article.^4^ In studies where longitudinal data were available, we used data from every visit.

### Ethics

MODEP received ethical approval no. 46/2010BO1 of the Medical Ethical Board of the University of Tuebingen. ABC‐PD received ethical approval no. 686/2013BO1 of the Medical Ethical Board of the University of Tuebingen. For DeNoPa, institutional review board approval was obtained from the Ethikkommission der Hessischen Landesärztekammer in Fankfurt, Germany (FF89/2008) on 26 March 2009. EPIPARK received ethical approval no. 09‐069. Oxford Discovery received ethical approval reference no. 10/H0505/71 from the NRES committee, South Central, Berkshire Ethics Committee.

### Statistical Analysis

To convert MoCA score to MMSE score, we used exactly the same equipercentile method with log‐linear smoothing that is described in the van Steenoven and colleagues article^4^ (see another work^3^ for methodological details). This method matches scores on the two tests by their percentile ranks after smoothing the distribution. The analysis was performed in the R statistical software (R Foundation for Statistical Computing), using the equate library.^11^ We then validated our MoCA to MMSE conversion and the proposed MoCA to MMSE conversion from the van Steenoven and colleagues article within each of our five cohorts and also across the five cohorts. This was carried out by calculating the difference (delta) between the true and equivalent MMSE and reporting the mean, standard deviation (SD), median, and interquartile range (IQR) of this delta along with the root mean squared error (RMSE). Smaller (in terms of absolute value) mean, SD, median, IQR, and RMSE denotes a more accurate conversion from MOCA to MMSE. We also calculated the intraclass correlation and the percentage of observations which were within ±2 points of the true and equivalent MMSE to enable comparison with a previous validation.^5^

## Results

We analyzed data from 1,161 individuals contributing 1,112 observations at baseline and 979 observations at follow‐up visits. (MODEP: 40 individuals with 39 observations at baseline, 40 at the second visit, 32 at the third and fourth visits, 25 at the fifth visit, 22 at the sixth visit, 26 at the seventh visit, and 20 at the eighth visit, giving 236 observations in total. The ABC‐PD study contributed 91 observations from 91 individuals. The DeNoPa cohort had 123 individuals with 93 observations at baseline and 121 at the second visit, giving 214 observations. The EPIPARK cohort had 112 individuals contributing 111 observations at baseline. The Oxford Discovery cohort had 795 individuals, of which 778 had baseline observations, 468 with visit 2 and 193 with visit 3 data, giving 1,439 observations in total.)

The MoCA adjusted scores ranged from 8 to 30 in our five cohorts with a median of 26 and an IQR range of 23 to 28. The mean MoCA was 25.0 and the SD 3.5. MMSE scores ranged from 13 to 30 with a median of 28 and an IQR of 27 to 29. The mean was 27.6 and the SD 2.3. These results indicate that MoCA may be better able to differentiate the range of cognitive function and is less prone to ceiling effects.

Table 1 shows that the conversion using the method proposed by van Steenoven and colleagues and our own conversion are remarkably similar with the equivalent MMSE only differing by 1 in 11 of 26 cases (ignoring where the MoCA was 4 or lower). However, it should be noted that we were both extrapolating to MoCA scores below 8 (and below 10 in the van Steenoven and colleagues article), so one must be cautious at the very low end of the distribution.

**Table 1:** Conversion from MoCA to MMSE using the equipercentile method with log‐linear smoothing using our datasets and compared to that from van Steenoven and colleagues

MoCA Total Adjusted	Equivalent MMSE Total (From the van Steenoven Article)	Equivalent MMSE Total (Internal Data Conversion)
1	6	1
2	9	2
3	11	4
4	12	10
5	13	13
6	14	14
7	15	15
8	15	16
9	16	17
10	17	18
11	18	18
12	18	19
13	19	20
14	20	20
15	21	21
16	22	22
17	22	22
18	23	23
19	24	24
20	25	24
21	26	25
22	26	26
23	27	26
24	28	27
25	28	28
26	29	28
27	29	29
28	30	29
29	30	30
30	30	30

MoCA was adjusted for the years of education. Scores that are in the shaded boxes are derived from extrapolated data.

Table 2 shows the difference (both within and across cohorts) between the true and equivalent MMSE for the van Steenoven and colleagues article conversion and our own conversion. The median difference was 0 (IQR –1 to 1) for the van Steenoven and colleagues article across all studies and the median difference was also 0 within each study, except for the MODEP cohort where the median difference was –1. However, even within the MODEP cohort, the IQR was –1 to 0, still showing that at least 50% of the results are very close. The median difference was also 0 (IQR, –1 to 1) using our own internal conversion across all the studies. The median difference was also 0 within each study, except for the EPIPARK cohort where the median difference was 1.

**Table 2:** Validation of the MoCA to MMSE conversion using both the conversion from the van Steenoven and colleagues article and our own internal conversion

		van Steenoven Article Conversion		Internal Conversion	
Cohort	N	MMSE Total–Equivalent MMSE: Mean (SD); Median (IQR)	RMSE	MMSE Total–Equivalent MMSE: Mean (SD); Median (IQR)	RMSE
MODEP^6^	236	–0.68 (1.45); −1 (−1, 0)	1.60	–0.29 (1.51); 0 (−1, 1)	1.54
ABC‐PD	91	–0.13 (1.74); 0 (−1, 1)	1.74	0.29 (1.80); 0 (−1, 1)	1.82
DeNoPa^7^, ^8^	214	0.08 (1.69); 0 (−1, 1)	1.69	0.53 (1.72); 0 (−1, 1)	1.80
EPIPARK^9^	111	0.32 (2.19); 0 (−1, 2)	2.21	0.74 (2.14); 1 (−1, 2)	2.25
Discovery^10^	1,439	–0.49 (1.86); 0 (−2, 1)	1.92	–0.03 (1.85); 0 (−1, 1)	1.85
All data	2,091	–0.39 (1.83); 0 (−1, 1)	1.88	0.05 (1.84); 0 (−1, 1)	1.84

Across all cohorts, the RMSE was remarkably similar comparing the van Steenoven conversion to our own conversion, 1.88 compared to 1.84, respectively. Comparing within the cohorts, the van Steenoven conversion worked slightly better within the ABC‐PD study, the DeNoPa cohort, and the EPIPARK cohort (lower RMSE and smaller mean differences), whereas our own conversion worked slightly better within the MODEP and Oxford Discovery cohorts.

The van Steenoven conversion demonstrated an intraclass correlation coefficient of 0.66 (95% confidence interval [CI]: 0.64–0.69) between the true and equivalent MMSE. For the van Steenoven conversion, 11.1% of MMSE equivalent scores were more than 2 points higher than true MMSE, 83.8% were within 2 points, and 5.1% were more than 2 points lower. Our own conversion showed an intraclass correlation coefficient of 0.66 (95% CI: 0.64–0.68), with 8.1% of MMSE equivalent scores were more than 2 points higher than true MMSE, 83.2% were within 2 points, and 8.7% were more than 2 points lower.

## Discussion

Our replication of the analysis resulted in a very similar conversion table to the one in the van Steenoven and colleagues, even though our sample is 10 times larger (n = 2,091, compared to n = 197). Validation of the conversion table from the van Steenoven and colleagues article shows that it has very good characteristics, with 0 median and small IQR of the difference and a RMSE, which is almost as good as our own internal conversion across all cohorts. An internal validation will almost always be better than an external validation, which further demonstrates the validity of the van Steenoven conversion. These findings are similar to a previously reported smaller validation study.^5^

Our sample had a wider range of MoCA and MMSE performance than a previous publication (lowest MoCA value 8 points, as compared to 17 points), which greatly enhances the generalizability of this conversion table. However, we still did not have any participants with a MoCA less than 8. This limitation is probably not of high clinical relevance given that it is unusual to recruit subjects with such poor performance into research. For example, in the UK, ethical committees would prohibit recruitment of subjects who could not consent into research unless it was a therapeutic trial that may have patient benefit.

The equipercentile method has the strengths that it can deal with nonlinearity within scales and that the equated scores will always be within the range of possible scores. However, the method is limited because it can lead to an irregular distribution of scores. An alternative approach to combine data across studies would be to internally standardize data using a Z‐score or a T‐score^12^ method; however, these approaches do not take into account the difference in distributions or variability across the populations; hence, we favor the former approach.

These results provide independent replication and validation of a previous conversion table from MoCA to MMSE. The closeness in scores using either conversion table shows that this approach is useful in converting MoCA scores to MMSE scores and enables harmonization and meta‐analysis across cohorts to determine determinants of cognitive impairment or decline in PD cohorts. This work needs to be extended to other domains, such as olfaction, depression, and so on, and to incorporate other methods, such as item response theory, to enable researchers to apply cross‐scale conversions. This will greatly enhance the utility of existing research data and facilitate greater collaboration and shared analyses, leading to more robust research findings.

## Author Roles

(1) Research Project: A. Conception, B. Organization, C. Execution; (2) Statistical Analysis: A. Design, B. Execution, C. Review and Critique; (3) Manuscript Preparation: A. Writing of the First Draft, B. Review and Critique.

M.L.: 2A, 2B, 3A

M.K.: 1A, 1B, 1C, 3B

M.T.M.: 2A, 2C, 3B

B.M.: 1A, 1B, 1C, 3B

M.S.: 1A, 1B, 1C, 3B

I.L.‐S.: 1A, 1B, 1C, 3B

W.M.: 1A, 1B, 1C, 3B

E.‐J.V.: 1A, 1B, 1C, 3B

M.T.M.H.: 1A, 1B, 1C, 3B

D.B.: 1A, 1B, 1C, 3B

Y.B.‐S.: 1A, 1B, 1C, 2A, 3B

## Financial Disclosures

M.K. received funding by the German Research Foundation (KA 3179/2‐1), received funding from the German Ministry of Education (BMBF), and research for two unrelated projects (“MitoPD” and “DysTract”). B.M. has received independent research grants from TEVA‐Pharma, Desitin, Boehringer Ingelheim, and GE Healthcare and honoraria for consultancy from Bayer Schering Pharma AG, Roche, AbbVie, TEVA‐Pharma, Biogen and for presentations from GlaxoSmithKline (GSK), Orion Pharma, and TEVA‐Pharma and travel costs from TEVA‐Pharma. B.M. is a member of the executive steering committee of the Parkinson Progression Marker Initiative of the Michael J. Fox Foundation for Parkinson's Research (MJFF) and has received grants from the Federal Ministry of Education and Research (BMBF), European Union (EU), Deutsche Parkinson Vereinigung, MJFF, Stifterverband für die deutsche Wissenschaft, and has scientific collaborations with Roche, Bristol Myers Squibb, Ely Lilly, Covance, and Biogen. B.M. is listed as co‐inventor in a patent application to the U.S. Patent Office related to the quantification of alpha‐synuclein in biological fluids for the purpose of improved diagnosis. W.M. receives funding from the EU, the MJFF, Neuroalliance, and Janssen. He received speaker honoraria from GSK, UCB, Licher MT, and Rölke Pharma. M.T.M.H. has received a Parkinson's UK innovation grant “Biomarkers for Dementia and Cognitive decline in Parkinson's disease,” Parkinson's UK Monument Discovery Award “Targeting the earliest pathways to Parkinson's disease,” EU small‐ or medium‐scale focused research project (STREP) Award “Virtual, physiological and computational neuromuscular models for the predictive treatment of Parkinson's Disease‐NoTremor,” and MJFF Biomarker Grant “Development of potential diagnostic biomarkers in Parkinson's disease.” There are no conflicts of interest. D.B. served on the advisory boards of Novartis, UCB Pharma GmbH, and Lundbeck. She received grants from the MJFF, the BMBF, JPND, EU–Horizon 2020, dPV (German Parkinson's disease association), Center of Integrative Neurosciences, Internationale Parkinson Fonds, Janssen Pharmaceutica, TEVA‐Pharma GmbH, and UCB Pharma GmbH and received honoraria from UCB Pharma GmbH, TEVA, and Lundbeck. There are no conflicts of interest. Y.B.‐S.– has received a Parkinson's UK innovation grant called “Biomarkers for Dementia and Cognitive decline in Parkinson's disease,” a Parkinson's UK grant called “Targeting the pathological pathways to Parkinson's (programme renewal),” an MRC grant called “FEMORAL: Factors affecting mortality, morbidity and patient outcomes after joint replacement surgery,” an NIHR grant called “CLAHRCwest (Collaboration for Leadership in Applied Health Research and Care for the West Country),” and NIHR‐MRC grant called “Reducing pathology in Alzheimer's Disease through Angiotensin TaRgeting—The RADAR Trial” and a British Heart foundation grant called “The influence of ambulatory central pressure in determining cardiovascular risk.”
